# When Does Stress Build Strength? Linking Stress Physiology to Resilience in the Age of Rapid Environmental Change

**DOI:** 10.1002/bies.70184

**Published:** 2026-09-28

**Authors:** Frederic Angelier, David Costantini, Cecilia Houdelier, Valeria Marasco

**Affiliations:** ^1^ Centre d'Études Biologiques de Chizé (CEBC), UMR 7372 CNRS La Rochelle Université Villiers‐en‐Bois France; ^2^ Department of Ecological and Biological Sciences University of Tuscia Largo dell'Università snc Viterbo Italy; ^3^ CEEC (Centre d’étude en Ethologie et Cognition) Rennes Univ, Caen‐Normandie Univ CNRS Rennes France; ^4^ Research Institute of Wildlife Ecology, Department of Interdisciplinary Life Sciences University of Veterinary Medicine, Vienna Austria

**Keywords:** abiotic stress, biology, biomedicine, climate change, environmental change, psychological resilience, stressor

## Abstract

The rapid, unprecedented rate of worldwide pressures organisms are currently exposed to, from climate instability to pollution, urges to expand research focus toward stress resilience. Here, our main goal is to call for an integrative effort to understand conserved processes underlying whole‐organism stress resilience with a specific focus on the following questions: (i) How can stress resilience be defined? (ii) What are its main underlying metrics at the whole‐organism level (focus on glucocorticoid hormones, oxidative stress, neural and behavioral metrics)? (iii) What is the role of stress inoculation‐ and hormetic‐like effects to build resilience throughout an organism´s lifespan? By building up on emerging knowledge spanning from behavioral biology and ecophysiology to biomedicine, we explore conserved mechanisms of stress resilience that are relevant for both environmental and human health. Finally, we encourage for more research on wildlife study systems, which experience natural and cumulative stressors across ecologically relevant settings, and can thus help uncover functional mechanisms of stress resilience across vertebrates, including humans.

## Graphical abstract

Environmental change exposes organisms to repeated and prolonged stressors. Stress history and context shape interacting glucocorticoid, oxidative, neural, and behavioral responses, producing variation in whole‐organism stress resilience and fitness‐related outcomes. Wildlife studies can reveal conserved vertebrate mechanisms relevant to environmental, population and human health.

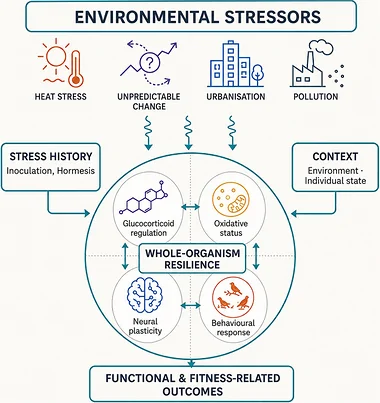

## 1 Introduction

Is stress more about disturbances in the external world—the stressors—or the resulting responses of these perceived stimuli in the body—the stress response/s? A long debate on the question of what stress is that has largely lost momentum (see Table 1 for main definitions). It encompasses both the above and likely even more—“*Let a thousand flowers bloom, but just remember to define your particular flower in the Methods section*,” as nicely put by Robert Sapolsky [1]. Here, we use stress to broadly indicate a dynamic state of homeostasis being challenged (systematically or locally; acutely or chronically), rather than only the stimulus (the stressor) or the stress response [2]. For clarity, however, we use the term stressor to refer to exposure to an external or internal challenge with the potential to disrupt homeostasis, and the stress response to refer to the regulated physiological and behavioral responses elicited by the perception of that challenge.

**TABLE 1:** Summary of the main definitions of stress and their key conceptual contributions, illustrating the evolution of the stress concept.

Author (s)	Definition	Key concept
Cannon (1932) [146]	Stress results from external or internal disturbances that threaten homeostasis, triggering coordinated physiological responses (the “*fight‐or‐flight*” response) to restore internal stability	Building upon Claude Bernard´s concept of the *milieu* intérieur, introduces the concept of homeostasis, establishing the physiological basis of stress responses
Selye (1956) [147]	Stress is the nonspecific response of the body to any demand placed upon it	Stress is viewed as a generalized physiological response, regardless of the nature of the stressor
McEwen & Wingfield (2003) [10]	Stress arises when environmental demands exceed the regulatory capacity of an organism, activating allostatic mechanisms to maintain stability through change	Introduces the concept of allostasis and allostatic load, highlighting that stress responses are adaptive but can become costly when prolonged or excessive

It is well acknowledged that acute/short‐term stress responses are generally adaptive and evolved to help animals cope with immediate life‐history challenges/stressors such as predator exposure, reduced resources, social subordination, or exposure to extreme climatic events [3, 4]. The sustained, repeated, or chronic activation of these systems, however, especially when experienced during early development, can incur physiological and fitness costs. Many studies in humans and other animals have shown clear deleterious consequences of such chronic activation, including disruptive effects on brain functioning, lifelong mental and metabolic disorders, and reduced longevity [5]. Despite this, a growing number of studies in wildlife and nonmodel organisms also show a large heterogeneity in the health and fitness consequences of chronic stress exposures not only across species, but also, and most interestingly, among individuals of the same species [1]. Evidence increasingly suggests that the phenotypic impacts of exposures to stressors, even when experienced repeatedly and/or over prolonged time periods, may be beneficial for organismal health and/or fitness outcomes. Whether it does so likely depends on interactions among genetic, developmental, and trans‐generational life‐history features, as well as stressor type, intensity, frequency, and duration [6, 7, 8]. Earlier concepts such as allostasis and the reactive scope model, have proved excellent conceptual frameworks to describe the costs of chronic stress and the main physiological basis of stress vulnerability [9, 10]. However, they fall somewhat short in accounting for the possibility that certain challenging exposures may promote adaptation (but see [11]).

A promising conceptual framework to shed light on this complexity and help broaden our understanding of organismal outcomes following stressor exposure, from pathology to adaptation, is that of resilience. Despite the broad field of resilience rapidly evolving, there is not yet a general consensus on how the concept should be defined. In biology, the concept is often defined as the ability of cells, tissues, and whole organisms to return to an original state following perturbations [11, 12, 13]. At its core, it refers to the process of restoring homeostasis, even when post‐stress physiological baseline values might differ from pre‐stress values [14]. Definitions of resilience can vary across disciplines such as behavioral sciences, ecology, neuroscience, physiology, and biochemistry, reflecting differences in levels of organization, time scales, and outcomes of interest [15]. Nevertheless, stress resilience is often broadly understood as the process of “achieving a positive outcome in the face of adversity” [16, 17]. Thus, a unifying feature of the concept is the coexistence of adversity/disturbance/challenge—irrespective of whether this is acute, repeated, or chronic—and the maintenance or recovery of functioning despite the exposure, reflecting an outcome‐based framework [18].

Resilience is often used interchangeably—sometimes erroneously—with the concept of resistance, or tolerance [15, 19]. Resistance can be broadly defined as the ability to persist despite a challenging event; whereas tolerance describes how much a host can prolong its survival or recover its reproductive success when facing an infection (reviewed in [19]). Resilience might determine the extent to which an organism, or a biological system, expresses resistance, or tolerance when faced with challenges. For example, in *Drosophila melanogaster*, Paolo et al. [20] show that experimental evolution under oral infection with *Pseudomonas entomophila* produces lines that are more resilient (i.e., recovering more effectively from infection‐associated harm), more resistant (i.e., with heightened ability to reduce bacterial loads), and more tolerant (i.e., with less damage for a given infection intensity). Therefore, resilience may be seen as a broader concept, within which tolerance and resistance represent two complementary mechanisms underlying organismal resilience, though this framework cannot be generalized [13].

Here, we seek to discuss the framework of resilience in the context of physiological stress responses. To date, research on resilience has mostly focused on the study of organismal responses to certain and often short‐term/acute stressors. Much less attention has been paid to how individuals exposed to repeated and longer‐term stressors might calibrate their behavior and physiology to better cope, or even thrive, under disturbance. This could happen through the ability of individuals to reinstate homeostasis through phenotypic plasticity, or conditioning “memory‐based” mechanisms enabling them to establish a new adaptive state [13, 21]. Such adjustment capacity is in accordance with the concept of Hypothalamic‐Pituitary‐Adrenal (HPA) flexibility, whereby individuals modify HPA axis regulation in response to environmental conditions, developmental stage, and prior experience, such that the same stressor might elicit different responses over time [22]. Here, we first review metrics of stress resilience that have been suggested to function at the whole organism level resolving around signals and consequences of the brain–body crosstalk (focus on glucocorticoid hormones, oxidative stress response signaling, as well as neuronal and behavioral metrics). Across diverse vertebrate species, these metrics have been linked to variation in key life‐history traits, including patterns of growth, survival rates and reproductive investment, thus ultimately influencing health and/or fitness outcomes (e.g., [23, 24, 25]). We will then specifically highlight emerging knowledge focusing on the role of mild/moderate environmentally generated stress exposures experienced over the life course. We will review how they can influence stress resilience, and to which extent such effects are long‐lasting, age‐ and context‐dependent. Finally, we will explore future research directions in the field and discuss how the study of wildlife responses to climatic and Anthropocene‐related environmental exposures—recently named as “bio‐exposome”—could provide key and novel opportunities to test hypotheses in real‐life settings moving beyond traditional models [26].

## 2 Metrics to Estimate Whole‐Organism Stress Resilience Across the Brain‐Body Cross‐Talk

### 2.1 Glucocorticoids

When coming to stress, most studies focus on the suite of behavioral and physiological changes that aim to restore homeostasis following a stressor [9, 10]. Most of these changes are mediated by neuroendocrine pathways that govern not only the immediate “*fight or flight*” response, but also the slower “*take it or leave it*” response [3]. The “*fight or flight*” response is mediated by the sympathetic branch of the autonomic nervous system [27], and despite its importance, it has been relatively overlooked because of the difficulty to measure its mediators (e.g., epinephrine), especially in wildlife. In contrast, most research has focused on the “*take it or leave it*” response, which is mediated by the Hypothalamic‐Pituitary‐Adrenal or Interrenal axis (HPA or HPI axis) and the secretion of glucocorticoids (GC) [28]. Indeed, GC are known to act on multiple physiological and behavioral systems [4, 29, 30], and above specific concentrations, to trigger an Emergency Life History Stage (ELHS) that aims to promote immediate survival at the expense of other traits that are not essential to immediate survival [3, 31].

Numerous theoretical and empirical studies have examined the intensity of the glucocorticoid stress response (i.e., how GC levels increase when a stressor occurs [3]), and how this stress response is modulated by the nature, the intensity, and the type of stressors [10, 32, 33]. Based on these results, the standardized glucocorticoid stress response has been extensively used to test when and to what extent individuals can resist stress by blocking the HPA axis and limiting the secretion of the hormone themselves [31, 34]. Numerous studies have, for example demonstrated that breeding individuals are often resistant to stress, especially when the fitness value of the current reproductive event is high relative to their residual reproductive value (the “*brood value hypothesis*”, [35, 36]). They have also reported that developmental conditions can have long‐lasting effects on the sensitivity of the organism to stress during adulthood (e.g., [37, 38, 39, 40]).

Surprisingly, GC and the HPA axis have been far less studied in the context of stress resilience. However, the termination of the GC stress response should logically be involved in the ability of individuals to recover from stressors, and therefore, to cope with them [41]. To recover from a stressor, circulating levels of GC first need to decrease down to levels that allow the organism to leave the ELHS and to resume normal activities. In addition, the duration and rate of this decrease in GC levels also determine post‐stress GC levels that can be slightly different from pre‐stress GC levels. These post‐stress GC levels may have some implications on the ability of the organism to cope with further stressors, notably through the permissive and preparative effects of GC on physiological and behavioral systems [4, 42]. At the mechanistic level, this GC stress recovery is determined by the rate of GC decrease once the stressor stops. This rate depends not only on the cessation of GC secretion by the HPA but also on the turnover of GCs in the bloodstream. The cessation of GC secretion is mainly governed by the activation of low‐affinity GC receptors by elevated GC levels while the turnover of GCs is mainly controlled by hepatic enzymes [4]. Because of the difficulty to study this post‐stress decrease of GC levels in natural settings [43], this stress recovery has classically been studied by using standardized biomedical protocols, which involve the injection of dexamethasone (DEX, a synthetic GC—e.g., [44, 45]). DEX binds to and saturates GC receptors, stimulating therefore GC negative feedback that inhibits the secretion of GC by the HPA axis [46, 47]. This protocol allows ecophysiologists to assess how fast GC levels decrease, and is therefore recognized as a relevant metric of the resilience of an organism to a stressor [45, 48].

At the ultimate level, there is growing evidence that the negative feedback can be tightly linked with the behavioral and physiological resilience to stress [48], but see [49]. Additionally, a few studies have reported that it can be linked with performance, such as reproduction (e.g., [41, 45]) and survival (e.g., [50]). This supports the idea that GC stress recovery, as the well‐studied GC stress response, could mediate life‐history decisions, such as the trade‐off between reproduction and survival [41, 51]. The assessment of post‐stress GC levels can also help in understanding how stressful experience may prepare or constrain the organism to the subsequent occurrence of an additional stressor; although other components of the HPA axis may also be crucial to consider, such as GC receptors, cochaperone binding protein, and FKPB5 [52, 53, 54]. Overall, these studies support the idea that GC stress recovery is a crucial metric to properly evaluate the ability of organisms to cope with stressors [45, 55], and including this overlooked metric in the study of the stress response and resilience appears crucial to obtain an integrative measure of how HPA flexibility can help individuals cope and recover from stressors [22].

### 2.2 Markers of Oxidative Status

Markers of oxidative status have increasingly been used to quantify the physiological consequences of stressor exposure and may therefore provide useful measures of stress resilience [6, 56]. But why should we focus on oxidative status? Oxidative status reflects the dynamic interaction between pro‐oxidant molecules—capable of oxidizing and thereby damaging biological macromolecules—and antioxidant defenses that mitigate or even repair such damage [57]. This interplay between pro‐oxidants and antioxidants is a key component of physiological homeostasis in all organisms, from plants to animals. Furthermore, oxidative processes not only reflect cellular damage and repair mechanisms but can also participate in physiological signaling across tissues, including neuroendocrine and neuroimmune pathways involved in brain‐body cross‐talk and blood‐brain barrier [58].

Growing evidence suggests that individual variation in cellular oxidative status might have organism‐level consequences, influencing traits such as growth, development, reproductive strategies, and lifespan [24]. However, a critical, yet understudied aspect, is how individuals differ in their ability to return to baseline oxidative status following a physiological or environmental challenge. This represents an important gap in the literature, as such information could improve predictions about an organism's capacity to cope with and recover from environmental stressors, and how such capacity might change over the life course (see paragraph below). For example, resilience to pollutant exposure in embryos of the Antarctic sea urchin (*Sterechinus neumayeri*) reflects maternal antioxidant provisioning [59]. In other words, offspring of mothers with high antioxidant levels inherit an enhanced resilience capacity to buffer pollutant‐induced oxidative damage early in life.

Despite this growing interest in oxidative status, there is currently no consensus on standardized metrics for its quantification [60]. This is partly due to the substantial heterogeneity in the way oxidative status is being regulated across tissues and taxa. Because oxidative status is often assessed in blood, especially in wildlife, it is important to recognize that blood‐based markers may not necessarily mirror oxidative processes in other tissues. In general, however, correlations between markers measured in different tissues tend to become stronger when an organism is under physiological challenge, making most oxidative markers measured in blood adequate proxies of systemic responses to environmental stressors (e.g. [61, 62, 63]). Furthermore, some molecular targets of oxidative status are evolutionarily conserved and provide promising avenues for cross‐species comparisons. Among these, antioxidant enzymes stand out due to their well‐characterized roles in reactive oxygen species (ROS) detoxification, their evolutionary conservation, and the feasibility of measuring both their activity and the expression of their encoding genes [57].

A notable example is the mitochondrial isoform of superoxide dismutase, SOD2, which protects cells from superoxide radicals generated within the mitochondria. Its function is conserved across a wide range of organisms, from fungi to humans and insects. Compared to the so‐called “dietary antioxidants”, the quantification of endogenous antioxidant enzymes is often preferable, as their expression and activity are under tight physiological regulation and are directly involved in maintaining cellular redox homeostasis [57]. Redox regulation, in turn, contributes to stress responses and may therefore represent one component of resilience [64]. Glutathione is another key antioxidant molecule owing to its high reactivity with ROS, its role as a cofactor for glutathione peroxidase, and its tightly regulated intra‐ and extracellular concentrations [57]. Changes in reduced glutathione, oxidized glutathione, or their ratio can provide information about cellular redox balance and the antioxidant response to stressor exposures [65, 66].

Oxidative damage represents another critical aspect of cellular stress and encompasses a broad range of biomolecules that undergo oxidative modification by ROS. Much research has focused on the deleterious effects of ROS on DNA, especially telomeric DNA, which are linked to accelerated aging, increased mortality, and overall heightened organismal vulnerability [67, 68, 69]. However, certain oxidative lesions, such as single‐base modifications, can be efficiently repaired by specific enzymatic mechanisms (e.g., base excision repair that generates 8‐hydroxy‐2’‐deoxyguanosine (8‐OHdG) as an intermediate). Similarly, specific enzymes can repair telomeric DNA and can be linked to stress resilience (i.e., telomerase) by reversing the impact of stressors on this aging mechanism [70]. The efficiency of such repair processes has been linked to stress resilience and extended lifespan [71, 72]. For example, in Caenorhabditis *elegans*, long‐lived mutants with enhanced DNA repair capacity and resistance to oxidative stress also exhibit increased survival and delayed aging [72]. By contrast, other forms of oxidative damage, including protein carbonylation, are generally irreversible because the introduction of carbonyl groups into proteins tends to cause structural and functional impairments that cells cannot easily repair or reverse. Consequently, the biological consequences of oxidative damage are highly context‐dependent, reflecting both the molecular targets affected, the magnitude and duration of the damage, and the organism´s capacity for repair. This context dependence makes redox balance difficult to quantify and highlights the need for repeated measurements of multiple oxidative status markers before, during, and after stressor exposure, and preferably linked to functional and fitness outcomes.

The interpretation of oxidative status metrics should also consider the possibility that antioxidant upregulation represents an adaptive resilience mechanism. For example, studies in *C. elegans* showed that mild challenges activate responses characterized by increased mitochondrial ROS signaling, activation of SKN‐1‐dependent transcriptional pathways (a functional ortholog of the mammalian Nrf2), and increased antioxidant capacity, which can lead to extended longevity [73, 74]. These responses can persist beyond the initial exposure and increase resilience to subsequent stressors through hormesis (see §3.2). Therefore, oxidative status metrics may reflect not only current oxidative balance but also the organism´s history of adaptive responses to previous stressors, which is particularly relevant in the context of stress resilience.

### 2.3 Neural Activity

The brain is the central organ mediating both the perception of stress and the organism's adaptive responses to stressors, making it essential for assessing stress resilience at the whole‐organismal level [16]. The hippocampus plays a key role in this context, as both neurogenesis and dendritic spine density are often suppressed by a variety of chronic stressors, including post‐traumatic stress disorders or social stress exposures [75, 76, 77, 78]. Stress‐induced alterations of hippocampal structure and function can be especially persistent when stressors occur during early development [79], often resulting in long‐lasting impairments in memory and learning [80].

On the other hand, the brain is not only vulnerable to stress exposure but also capable of pronounced structural remodeling depending on context, duration, and intensity of exposure [1, 79]. For example, studies in human sport physiology show that physical activities such as running—which transiently elevate circulating GC—are associated with enhanced hippocampal neurogenesis and improved dendritic architecture [81]. Similarly, in rodent models, increased environmental complexity through enrichment has been shown to promote hippocampal neurogenesis and structural plasticity [82, 83].

Molecular signatures associated with resilience can also be detected within specific brain regions. Transcriptomic approaches, which quantify genome‐wide gene expression profiles in sampled regions or cell populations, have highlighted regulatory networks that could help to distinguish stress‐resilient from more vulnerable phenotypes. For example, Gray et al. [84] demonstrated that wild‐type mice subjected to 21 days of chronic restraint stress and then exposed to a novel acute stressor (a forced swim test) activated more distinct hippocampal gene networks compared to naïve mice (mice exposed only to a forced swim test). Such effects were partially mitigated in the group of mice that were allowed to recover 21 days after the chronic stress exposure before being exposed to the acute stressor. However, within these “recovered mice”, the hippocampal expression of many stress‐induced genes (part of the NF‐kB signaling cascade) remained altered and did not return to baseline. These apparent persistent changes indicate some form of memory‐based mechanism that brought these animals to potentially “new homeostatic set‐points” through molecular reprogramming. Transcriptome profiling of defined brain regions across individuals with different developmental and later‐life experiences in respect to adversity can therefore provide mechanistic insights into why some brains maintain function under challenging circumstances while others deteriorate [85].

Although analyses of brain tissues require terminal sampling, RNA‐Seq approaches now enable transcriptome profiling from minimally invasive tissues such as blood. While blood transcriptome profiling does not directly reflect brain‐specific molecular activity, it could help to identify non‐invasive biomarkers associated with neural stress resilience [86]. Blood transcriptomics allows longitudinal monitoring of the same individuals and has been applied in non‐model and wildlife species to study responses to environmental changes, such as urbanization (e.g., [87, 88]). Nevertheless, complementary experimental studies in the lab will remain essential for disentangling causal mechanisms and validating molecular pathways that cannot be directly investigated through peripheral transcriptomics alone. Moreover, advances in noninvasive neuroimaging, such as functional Magnetic Resonance Imaging, have enabled longitudinal tracking of stress‐induced changes in large‐scale brain networks connectivity, revealing how and also when resilience emerges at the whole‐organism level [89, 90]. Although these approaches are currently restricted to laboratory settings, they can be applied to wildlife species in captivity, thereby potentially helping to bridge the gap between laboratory and wildlife research (e.g., [91]).

### 2.4 Behavioral and Cognitive Metrics

The resilience of an individual from a behavioral perspective can be assessed using different time scales and methodologies. Short‐term assessments compare behaviors before and after stress exposure to evaluate recovery capacity. A rapid return to baseline, whether in terms of locomotion, exploration, feeding, or maintenance behaviors, is generally associated with greater resilience [92]. Similarly, rapid recovery of behaviors involving high levels of control (i.e., expectation, anticipation) can reveal cognitive resilience capacities [93]. Vocalizations following challenging exposures could also provide new markers of individual resilience [94, 95]. For example, Scopa et al. [96] proposed that the rapid decline in snoring, a stress‐releasing behavior in horses, may indicate behavioral resilience to an acute novel‐object challenge. Similarly, adaptive behaviors such as increased water intake or shade seeking during heat stress may contribute to resilience by limiting physiological disruption [97]. However, their expression alone does not demonstrate resilience, as this requires evidence that the behavior is associated with the maintenance or recovery of relevant organismal functions. Furthermore, the extent to which a behavioral response provides information about stress resilience is likely to depend on stressor type and intensity. For example, while self‐grooming behaviors may indicate that the animal is returning to a state of homeostasis, they may also reflect vulnerability and reduced stress coping capacity [98]. While behavioral responses to acute stress can be used to characterize short‐term recovery of homeostasis, repeated behavioral measurements during prolonged stress exposure can be used to quantify the magnitude and persistence of changes (involving activity patterns, social or reproductive behavior), and thus an organism´s overall ability to be minimally affected (i.e., low sensitivity) by environmental adversity. These responses can lead to the identification of resilient or susceptible profiles through assessment of depressive‐like behaviors (i.e., anhedonia, social motivation) anxiety, and cognitive bias (pessimistic vs optimistic) [99].

Analyzing the relationships between individuals' personality traits or coping styles (i.e., consistent among‐individual differences in behavior across time and contexts; for coping style, these behavioral differences are correlated with physiological stress responses [100]) and their resilience can help predict variation in the capacity to respond to environmental exposures, especially in the context of repeated or prolonged exposures. Indeed, in a number of species, individuals that are bold, fast explorers, aggressive toward congeners, neophiles, or generally express a proactive profile, typically show greater resilience in situations of acute or chronic stress (e.g., [101, 102]). However, the relationships between personality/coping style and stress resilience may be context‐dependent. For example, in rats, proactive individuals are less sensitive to food deprivation but more vulnerable to social stress, which can compromise their social status [100]. This highlights potential trade‐offs between resilience‐related traits. Thus, the relevance of personality or coping style assessment lies in identifying context‐dependent patterns of resilience and vulnerability to various stressors. The work of Lambert et al. [103] also shows the importance of exploring the continuum of personalities that exist between proactive and reactive individuals. Flexible individuals (using alternatively proactive or reactive strategies) show greater resilience than those with extreme coping styles. This suggests that behavioral flexibility, defined as the ability of an individual to respond directly to and adjust their behavior in response to environmental stimuli [104], could also be a relevant indicator in the assessment of individual stress resilience. Indeed, recent work highlights its fundamental role in the processes of colonizing new environments, and adapting to anthropogenic pressures [105].

The environmental context can also modulate stress resilience. In social species such as rodents or primates, affiliative interactions may act as a buffer against stress exposure [106], for instance by attenuating physiological and behavioral responses to stressor exposure and promoting faster recovery. Moreover, in many species, environmental enrichment can increase individuals' ability to cope with environmental disturbances [107]. It thus appears that behavioral stress resilience is complex and context‐dependent, likely reflecting behavioral syndromes rather than single traits. Combining behavioral and physiological markers seems therefore necessary to identify resilient phenotypes [94].

## 3 Evidence on the Role of Phenotypic Plasticity to Build Stress Resilience

### 3.1 Developmental Plasticity and Stress Resilience

Environmental conditions during early development can have profound and long‐lasting effects on physiology and behavior, ultimately influencing adult health. Earlier epidemiological evidence in humans showed that adverse developmental conditions—such as reduced body weight at birth and various prenatal stress exposures—are associated with increased risk of morbidity and mortality later in life, providing the empirical foundation for the “*Developmental Origins of Health and Disease Hypothesis*” [108, 109]. Within this framework, early‐life adversity was largely viewed as a constraint that negatively programs physiological systems, thereby having negative organismal outcomes. More recently, however, theoretical models and empirical evidence across vertebrates have transformed this unidirectional view, suggesting that adaptive phenotypic calibration can still occur under challenging exposures through developmental plasticity [7, 110]. For example, studies in mammals and birds suggest that moderate early‐life stress exposure can enhance later stress‐coping capacity and, at least to a certain extent, organismal performance, possibly via fine‐tuning HPA axis responsiveness, neural gene expression, and immunity [38, 111, 112, 113]. The “*Stress Inoculation Model*”, mostly employed in the field of psychoneurobiology research, posits that early‐life adversities that are not overwhelming but just challenging enough to stimulate cognitive and emotional processing improve stress coping strategies into adulthood [114]. According to this hypothesis, the relationship between early‐life stress exposure and organismal function follows a nonlinear, often described as inverted U‐shaped or J‐shaped relationship [115]. Peak performance and resilience are predicted at intermediate levels of stress exposure (Figure 1A), mediated through stimulatory and preparatory effects of candidate mechanisms, especially glucocorticoid signaling and neural plasticity [1]. Though beyond the scope of our review, we also acknowledge that stress inoculation might rely on several other mechanisms, such as cell‐autonomous “memory” ones, that extend beyond glucocorticoid pathways of resilience (e.g., see [116]).

**FIGURE 1:**
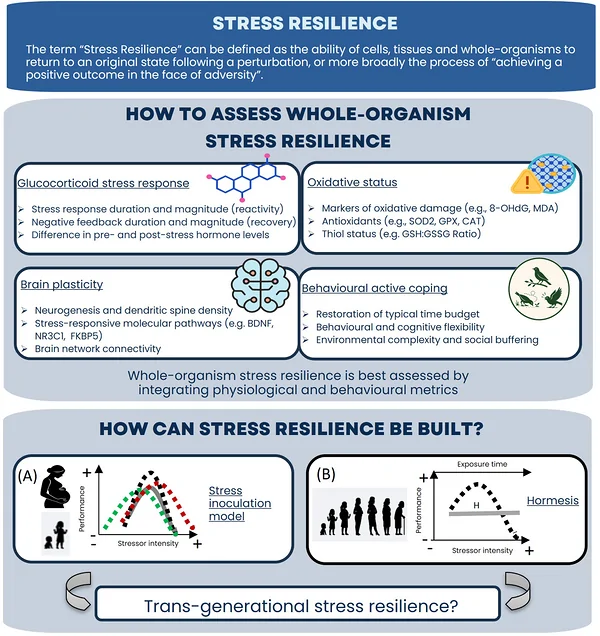
Infographic summary of the manuscript. In (A): moderate exposure to a stressor in early life can promote subsequent performance (gray solid line). Included in the graph are three hypothetical curves (dashed lines) describing how stress inoculation experienced during early life might influence performance into adulthood: (i) a leftward shift of the curve (green dashed line) might make lower levels of the stressor promote better performance; (ii) an upward shift of the curve (black dashed line) shows that inoculation might promote higher peak performance levels in response to the stressor; (iii) a rightward shift of the curve (dashed red line) shows that inoculation might enable maintaining peak performance levels at higher levels of exposure to the stressor. Modified after [148]. In (B): graphical representation of the hormesis concept in respect to a biphasic dose‐response relationship. Dose‐response curves showing the changes in performance in response to exposure level of a stressor over time in relation to a reference group (control group: grey). The hormetic zone (indicated with “H”) is the area under the curve and above the zero equivalent point (the point of the hormetic curve where performance equals that of the control). Modified after [121, 149]. Abbreviations: 8‐OHdG, 8‐hydroxy‐2'‐deoxyguanosine; MDA, malondialdehyde; SOD2, superoxide dismutase 2; CAT, catalase; GPX, glutathione peroxidase; GSH, reduced glutathione; GSSG, oxidized glutathione disulfide; BDNF, brain‐derived neurotrophic factor; NR3C1, Nuclear Receptor Subfamily 3 Group C Member 1 or glucocorticoid receptor (GR); FKBP5, FK506 Binding Protein 5. Graphical elements were downloaded from Canva.com.

While mild challenges could be beneficial, the stress inoculation model posits that excessive challenges are associated with suboptimal phenotypes, reflecting either insufficient developmental calibration or pathological dysregulation of developing systems [1]. Importantly, the shape and position of this nonlinear relationship is not only species‐specific but can vary within individuals of the same species depending on multiple factors, including the timing, duration, predictability of the stressor, as well as individual genetic background, and trans‐generational historical events or carry‐over effects [7, 8, 117]. Though yet to be tested, the adaptive value of such developmental calibration might also depend on the degree of environmental matching between early‐life and later‐life conditions. Under the Predictive Adaptive Response hypothesis, phenotypes calibrated during development are expected to perform better when adult environments resemble those experienced early in life, whereas mismatches reduce performance [110]. Together, these hypotheses predict that peak resilience would occur following mildly challenging exposures when early‐life conditions accurately predict future environments.

### 3.2 Hormesis: A Framework Linking Phenotypic Plasticity With Stress Resilience Over the Life Course

Because most of the resilience‐related physiological systems mentioned above are shaped during development, this stage is critical to study the resilience of organisms to change. After the developmental phase, these physiological systems are however, not fixed for the rest of the organism's life (i.e., the post‐developmental phase) and physiological plasticity can also occur later in life with potential impact on the resilience of adult organisms [118]. Although these late‐life changes are predicted to be smaller than those occurring during development, they could still allow an organism to adjust its physiology to the environmental conditions it is likely to encounter in the future. From an evolutionary perspective, such plasticity may be beneficial when environmental conditions change from early life to adulthood, thereby possibly buffering the consequences of developmental mismatches. It may then improve the resilience of an organism by dampening the impact of developmental events on physiological systems (e.g., GC [119]). For example, exposure to repeated mild stressors during adulthood is known to increase longevity in zebra finches [120], and this effect could potentially be mediated through an improved resilience of some physiological systems, such as oxidative status and HPA axis functioning [71, 120].

Such adaptive physiological plasticity during adulthood is however, expected to occur mainly under mild stressful conditions in line with the hormesis concept [121, 122]—a dose–response phenomenon in which low to moderate exposure to a stressor elicits stimulatory or beneficial effects, whereas high doses are inhibitory or toxic [123, 124] (Figure 1B). Theoretically, this concept can apply to both acute and chronic stress, although this may involve different mechanisms depending on stress duration and severity. Originally developed in toxicology and radiation biology, hormesis has since been documented across biological levels, from cellular stress responses to whole‐organism performance, and across taxa, including microbes, plants, invertebrates, and vertebrates [6]. Hormesis shares conceptual similarities with the Stress Inoculation model when it refers to a form of “conditioning” whereby exposure to a mild stressor enhances the capacity to cope with exposure to similar or higher levels of the stressor when encountered again on subsequent occasions. Contrary to stress‐inoculation‐like effects, hormetic conditioning could occur at any point during the life course, though it is expected to be stronger during early development [121]. Both frameworks emphasize that biological systems do not respond linearly to challenge intensity, and that optimal function often emerges at intermediate levels of exposure. Once stressors exceed the hormetic range—because they are too intense, too prolonged, or repeated without sufficient recovery—adaptive responses give way to maladaptive neurophysiological changes and reduced resilience to subsequent stressors [125]. Established examples come from the biomedical and psychological literature on post‐traumatic stress disorders (PTSD). PTSD is known to affect organismal oxidative status [126], the functioning of the HPA axis, and neural systems [127], all of which contribute to stress resilience (see §2). Importantly, there is increasing evidence that these PTSD‐related neurophysiological changes may affect the ability of the organism to further cope with stressors and its resilience to stressors [127].

Resilience (or the absence of resilience) can also be built through carry‐over effects, meaning that the events occurring during a specific phase of the annual life cycle can have some impacts on the resilience of organisms during subsequent stages [128]. These carry‐over effects may be mediated through the long‐lasting impact of stressful events on neurophysiological systems (such as those described previously, see §2), highlighting therefore that carry‐over effects can be closely linked to the memory‐based mechanisms that were described earlier. Numerous studies have reported that carry‐over effects are induced by nutritional conditions or infection status [e.g., [129]], two factors that are tightly linked to the mechanisms that probably mediate organismal resilience (e.g., glucocorticoids, oxidative status). However, we currently have limited evidence showing that resilience can be affected through such carry‐over effects, and future studies linking carry‐over effects, resilience, and its metrics (see §2.) are needed.

## 4 Future Prospectives

Addressing current knowledge gaps in stress resilience requires integrating environmental change as a central experimental dimension rather than treating it as background noise. Understanding how environmental conditions shape individual resilience‐related responses, and how these phenotypic responses scale up to influence population dynamics, species persistence, and ecosystem functioning remains a major research challenge [130, 131]. In this context, wildlife systems can provide compelling, yet underexploited, opportunities to investigate how organisms respond to repeated acute, chronic, and interacting stressors under ecologically realistic conditions. There is accumulating evidence showing that exposure to climatic and anthropogenic‐related pressures, ranging from urban noise, artificial light at night, chemical pollutants, and altered resource landscapes, can lead to profound variation in GC functioning and negative feedback efficiency, markers of oxidative status, neuronal re‐programming, and behavioral coping styles [132, 133, 134, 135]. Importantly, these responses are not uniformly maladaptive. For example, urban‐dwelling birds and reptiles frequently show blunted endocrine stress responses, or altered baseline hormone levels compared to rural conspecifics without an apparent reduction in organismal performance or fitness‐related outcomes [136, 137], suggesting possible physiological canalization rather than dysregulation. Understanding the extent to which phenotypic plasticity has a role in explaining such responses through inoculation‐like effects, or hormetic conditioning, is an exciting new research frontier (see also Figure 2).

**FIGURE 2:**
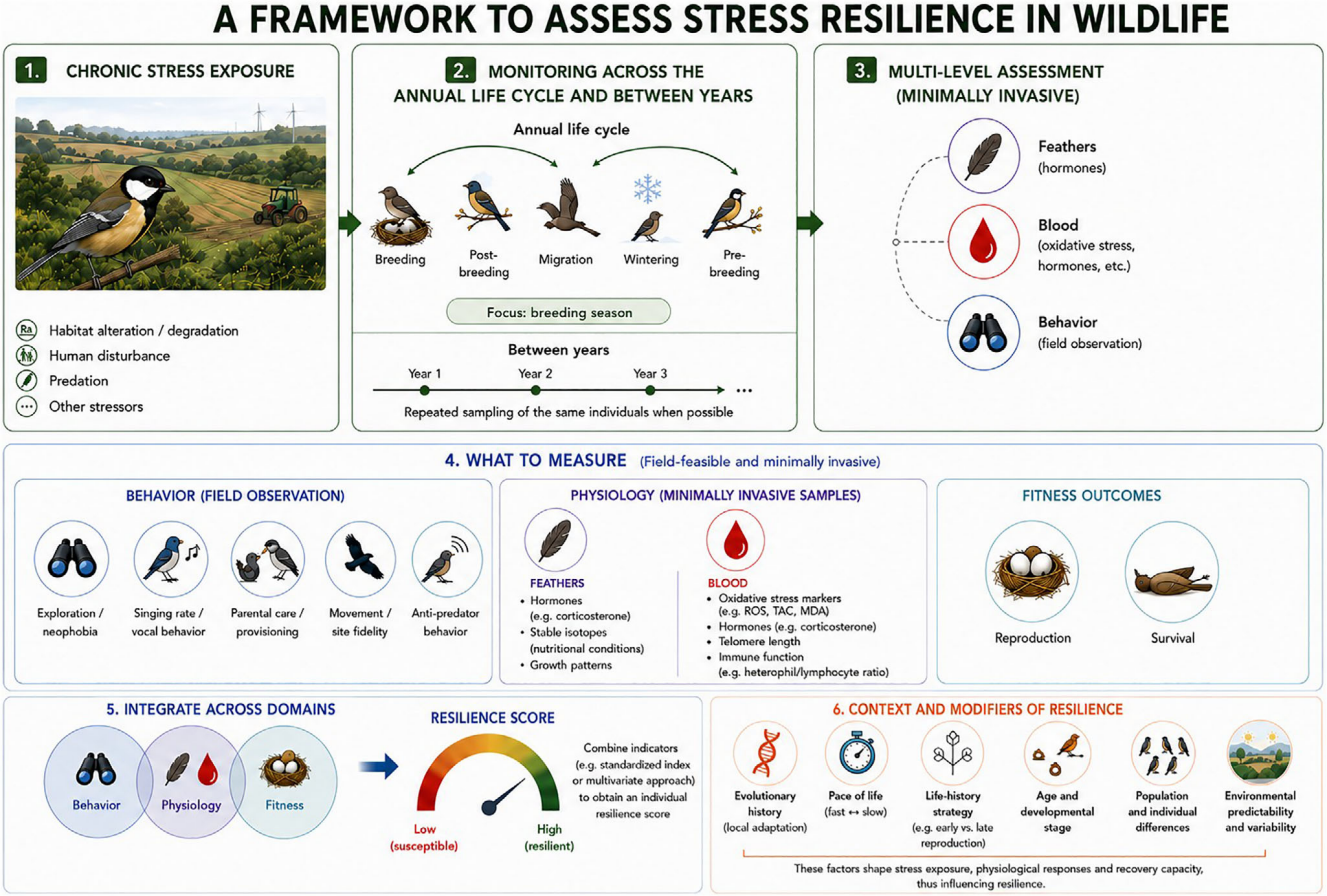
A conceptual framework for assessing stress resilience in wild birds exposed to chronic environmental stressors. Responses to disturbances are assessed by repeatedly monitoring individuals across the annual life cycle (1, 2). Resilience is quantified using field‐compatible measures of behavior and physiology, allowing individuals to be classified along a resilience‐susceptibility continuum according to their ability to maintain homeostasis and fitness under stress exposure (3). Whole‐organism resilience is assessed by integrating behavioral, physiological, and fitness‐related metrics (4, 5), including behavioral responses, feather‐ and blood‐based biomarkers, and life‐history fitness outcomes. The resulting integrative resilience score should be interpreted in light of key biological and environmental modifiers, including evolutionary history, pace of life, life‐history strategy, developmental stage, and environmental predictability (6). Schematic illustration created with the assistance of ChatGPT (OpenAI).

Studies increasingly examine how environmental conditions, especially through parental and early‐life effects, influence physiology and behavior across generations, extending classic concepts of phenotypic plasticity to multi‐generational contexts [138]. Accumulating evidence suggests that parental pre‐ and postnatal conditions can modulate the fitness of their offspring and drive their ability to cope with subsequent environmental challenges [138, 139, 140]. While most of the work on the inheritance of stress has traditionally focused on pathological outcomes of some chronic stressors [141], the possibility that resilience itself can be transmitted across generations remains largely unexplored. Nevertheless, studies in vertebrates provide relevant examples. In rats, natural variation in maternal care influences offspring HPA axis function, with greater maternal licking and grooming behavior being associated with increased hippocampal glucocorticoid receptor expression, enhanced negative feedback sensitivity, and attenuated corticosterone responses to acute stress [142]. These effects involve epigenetic modification of the hippocampal glucocorticoid receptor promoter and can themselves be transmitted behaviorally through maternal care [143]. In Japanese quail, prenatal exposure to corticosterone altered stress‐coping related traits in offspring, with offspring responses also depending on their own postnatal developmental environment [139]. These studies demonstrate intergenerational transmission of stress‐response phenotypes, although they do not establish the transmission of resilience itself, or germline epigenetic inheritance. Environmentally mediated epigenetic processes remain promising candidate mechanisms, as suggested for example in the transhormesis hypothesis [93]. In *C. elegans*, hormetic metabolic stress (through AMPK activation and mTOR suppression) is transmitted across generations via soma‐to‐germline histone‐dependent mechanisms [144]. Whether comparable germline mechanisms transmit resilience‐associated phenotypes in vertebrates remains an open question.

Finally, we acknowledge that resilience is not simply the absence or the opposite of vulnerability: the mechanisms that actively promote resilience may differ from those whose modulation reduces vulnerability as argued in Bhatnagar [17]. Differentiating these processes requires controlled experimental manipulations, which can be challenging outside the laboratory environment. The integration of whole‐organism metrics of resilience across behavioral and physiological domains (see Figures 1 and 2 for a list of potential metrics) could help overcome these limitations and strengthen interpretation. As resilience is both a dynamic process and an organismal outcome, a key challenge for advancing the field is to determine how its fitness consequences may vary among different relevant metrics that may jointly influence stress resilience at the individual level. This is because resilience in one physiological or behavioral axis might trade off against reduced resilience in others. When responses across multiple axes covary consistently, multivariate approaches could be used to derive a context‐specific individual resilience score (Figure 2). However, we also recognize the possibility that resilience may be better represented as a multidimensional “resilience mosaic”, in which some traits resist disturbance, others recover, and others remain persistently impaired. The relationships among these components are likely to depend on the stressor type, intensity, duration, and environmental context, and may arise from interactions among endocrine and cellular regulatory pathways, as well as variation in life‐history strategies [145]. An equally important challenge is to determine how resilience expressed at the whole‐organism level influences resilience at higher‐level outcomes, from populations to species and communities. Understanding the evolution of resilience will require identifying which underlying traits (or combinations of traits) generate consistent variation in fitness under defined environmental conditions [11], and are therefore likely to be under selection. The large variability in life history strategies in wild vertebrates, including within species and populations, offers powerful models to study resilience and thus for improving predictions of how natural populations cope with, or even thrive, under ongoing rapid environmental change.

## Author Contributions

F.A., D.C., C.H., and V.M. conceived the idea and wrote the manuscript.

## Funding

VM acknowledges support from the Austrian Science Fund (FWF) for preparation of this paper (grant number I 6634) and start‐up funds from the University of Veterinary Medicine, Vienna.

## Conflicts of Interest

The authors declare no conflicts of interest.

## Data Availability

No new data were generated or analyzed in support of this article.
